# Adsorption and Aggregation Behavior of Si, Sn, and Cu Atoms on Carbon Nanotubes (CNTs) According to Classical Molecular Dynamics Simulations

**DOI:** 10.3390/nano15181406

**Published:** 2025-09-12

**Authors:** Qiran Yuan, Qingshui Liu, Hui Li

**Affiliations:** Key Laboratory for Liquid-Solid Structural Evolution & Processing of Materials, Ministry of Education, School of Materials Science and Engineering, Shandong University, 17923 Jingshi Road, Lixia District, Jinan 250062, China; sweetdespot@163.com (Q.Y.);

**Keywords:** molecular dynamics simulation, carbon nanotube, silicon, tin, copper, nanoparticle

## Abstract

Using molecular dynamics (MDs) simulations with Materials Studio 8.0 software, we systematically investigated the adsorption and aggregation behaviors of silicon, tin, and copper atoms on the surface of (7,7) single-walled carbon nanotubes (SWCNTs). Silicon, tin, and copper were selected due to their distinct bonding characteristics—covalent (Si), semi-metallic (Sn), and metallic (Cu)—and their relevance in potential composite interface applications such as energy storage, thermal management, and electronics. The results indicate that silicon atoms form multi-layered concentric shells; however, the rigidity of their covalent bonds makes the resulting structures susceptible to disruption by local density fluctuations. Tin atoms form a limited number of stable concentric shells benefiting from the flexibility of their semi-metallic bonds. In contrast, copper atoms rapidly aggregate into disordered clusters due to their high diffusivity and metallic bonding. Within the confined geometry of the carbon nanotubes, all three types of atoms exhibit a tendency toward spiral growth, but their regularity depends on the properties of their chemical bonds, leading to distinct spiral features. These findings are further supported by linear density and radial distribution function (RDF) analyses.

## 1. Introduction

Carbon nanotubes (CNTs) have attracted significant research attention in the field of nanomaterials since their discovery by Sumio Iijima in 1991 due to their exceptional mechanical, electrical, and thermal properties [1]. When incorporated as a reinforcing phase into polymer matrices to form nanocomposites, CNTs can substantially improve the electrical, thermal, and mechanical performance of the resulting materials. In recent years, with the growing interest in carbon-based nanocomposites, these materials have demonstrated wide-ranging application potential in diverse fields, including sensors, energy storage and conversion, catalysis, quantum information processing, and microelectronics [2,3,4,5,6,7].

In composite materials, the structure and chemical states of the interface formed between the matrix and the reinforcing phase directly affect the mechanical, electrical, thermal, and other functional properties of the composites [8]. Heterogeneous nucleation represents a typical mechanism of adsorption and aggregation during the fabrication of composites. In this process, the reinforcing material often acts as an “impurity” relative to the matrix, providing nucleation sites and contributing to the stabilization of crystal nuclei [9,10,11]. The formation of a new phase typically begins at the surface of such impurities, whose properties significantly affect both the nucleation rate and the extent of supercooling during the early stages [12]. Similarly, in CNT/matrix composites, the interfacial characteristics between the CNT reinforcing phase and the matrix material are critical to determining the overall material performance [13]. Therefore, a comprehensive understanding of atomic-level interfacial interactions is essential for the rational design and optimization of composite materials.

Experimentally, studies on the interfacial bonding performance of CNTs/matrix composites primarily rely on micro-characterization techniques, Raman spectroscopy analysis, and nano-mechanical pull-out experiments [14,15,16]. Although data obtained through experimental methods are generally objective and reliable, limitations in current testing techniques often introduce data variability, leading to significant experimental errors and hindering direct observation of the actual adsorption and aggregation behaviors at the interface [17,18,19,20,21]. These challenges have significantly hindered the development of the CNT-based composites field. To address these limitations, molecular simulation methods offer a powerful approach for investigating the microscopic interaction mechanisms between CNT reinforcing phases and matrix phases in composites.

CNTs are often supplemented with metals to enhance their intrinsic properties, such as electrical conductivity, thermal stability, mechanical strength, and catalytic activity, enabling applications in energy storage, sensing, and nanocomposites. For instance, Xing et al. reviewed recent molecular dynamics studies on metal matrix composites (Cu, Al, Ni) reinforced with carbon nanomaterials such as CNTs [22], providing a detailed analysis of how CNTs’ characteristic layer number, volume fraction, diameter, and loading conditions, as well as interface interactions, influence composite performance. Parallel to metal matrix systems, numerous molecular dynamics studies have also been conducted on CNT/semiconductor matrix composites [23,24,25].

Silicon, as a cornerstone semiconductor, when composited with CNTs, significantly enhances lithium-ion battery anodes by alleviating volume expansion issues and boosting electrical conductivity, enabling high-capacity energy storage systems [26]. Sn/CNT composites similarly advance energy storage, providing improved cycle stability and high reversible capacity in batteries, addressing challenges in sustainable power technologies [27]. Cu/CNT composites excel in thermal management for microelectronics, offering superior heat dissipation and mechanical reinforcement, which is critical for next-generation interconnects and cooling solutions [28]. Moreover, these composites exemplify distinct CNT interface archetypes, interconnects, yhich are covalent bonding in Si/CNT (strong, directional interactions), semi-metallic in Sn/CNT (intermediate flexibility) and metallic in Cu/CNT (weaker, van der Waals-dominated) battery anodes, which were systematically cross-compared elucidate general principles of nanoscale atomic organization and interfacial dynamics [22,29].

Despite the focus on Si/CNT, Sn/CNT, and Cu/CNT composites to analyze distinct interface archetypes, many other useful metal–CNT composites exist but are not addressed in this study. For example, Fe/CNT composites are valuable for applications like magnetic reinforcement in steel and catalytic CNT synthesis [30], but they were excluded to prioritize the bonding gradation framework without ferromagnetic complexities.

Although the Si/CNT, Sn/CNT, and Cu/CNT composite interface systems have been extensively studied [3,4,5,6,7,8,9,10,11,12,13,14,15,16,17,18,19,20,21,22,23,24,25,26,27,28,29,30,31,32,33,34,35], research in the field of composites containing CNTs remains in its early stages, with the microscopic interaction mechanisms and governing laws at CNT/matrix interfaces yet to be fully elucidated, necessitating further in-depth investigation.

At the molecular dynamics simulation level, Diao et al. conducted a systematic investigation into the interfacial behavior between CNTs and Si atoms, highlighting the significant application potential of such interfaces in heat transfer [24]. Additionally, XING et al. reviewed recent molecular dynamics simulation studies on the strengthening effects of CNTs and graphene in metal matrix composites (e.g., Cu-based composites) [22]. Nevertheless, critical issues, such as the characteristics, differences, and underlying mechanisms of CNT-induced heterogeneous aggregation behaviors across these distinct systems, remain underexplored.

In summary, this study employs classical molecular dynamics simulations to systematically investigate the adsorption and aggregation behaviors of silicon, tin, and copper atoms induced by CNTs in a comparative manner. To the best of our knowledge, this is the first work to provide a unified and systematic comparison of Si/CNT, Sn/CNT, and Cu/CNT systems within a unified simulation framework. This study focuses on characterizing the layered growth patterns of concentric shells, along with the planarization, and fragmentation processes of different atomic species on the exterior of CNTs, as well as the distinct spiral growth phenomena exhibited by atoms within CNTs over the simulation timeframe. Based on these observations, combined with analyses of the system’s two-dimensional linear density and radial distribution functions, corresponding interpretations are provided. Overall, this work aims to provide theoretical insights for cross-scale material design and the development of functionalized composite interface materials for diverse applications.

## 2. Materials and Methods

### 2.1. Molecular Dynamics Simulation and Potential Model

Molecular dynamics simulation is an important computer simulation method for solving multi-body problems at the atomic and molecular scales. By solving the Newtonian equations of motion for all particles, it can simulate fundamental processes related to atomic motion trajectories, thereby capturing the dynamic characteristics of multi-particle systems. A key aspect of molecular dynamics simulation lies in selecting an appropriate potential model and employing an effective integration method for the equations of motion.

The COMPASS potential field (Condensed-phase Optimized Molecular Potentials for Atomistic Simulation Studies) is specifically optimized for condensed-phase applications and is particularly well-suited for simulating organic molecules, polymers, and certain inorganic small molecules. Reported research indicates that the COMPASS force field demonstrates reliable performance in modeling the interactions between CNTs and silicon, which can be attributed to its parameterization based on quantum mechanical calculations, thereby enabling more accurate representation of interfacial properties [36,37,38]. Therefore, in this study, the COMPASS force field with a valence state model was employed to describe the empirical potential energy between C-C, Si-Si, and Si-C atom pairs; its general form is as follows [36]:

$$E=E_{bonds}+E_{angles}+E_{torsions}+E_{inversions}+E_{cross}$$

Bonds:

$$E_{bonds}=\sum \frac{1}{2}\;K_{b}(r-r_{0}{)}^{2}$$

Angles:

$$E_{angles}=\sum \frac{1}{2}\;K_{\theta }(\theta -\theta_{0}{)}^{2}$$

Torsions:

$$E_{torsions}=\sum \frac{1}{2}\;K_{\phi }(1+\cos(n\phi -\phi_{0}))$$

Inversions:

$$E_{inversions}=\sum \frac{1}{2}\;K_{\chi }(\chi -\chi_{0}{)}^{2}$$

Cross Terms:

$$e.g.,\;\mathrm{bonds}–\mathrm{bonds}:\;E_{bb}=\sum K_{bb}(r_{i}-r_{i0})(r_{j}-r_{j0})$$

$$\mathrm{Bonds}–\mathrm{angles}:\;E_{ba}=\sum K_{ba}(r-r_{0})(\theta -\theta_{0})$$

Van der Waals:

$$E_{vdW}=\sum 4\epsilon \left[{\left(\frac{\sigma }{r}\right)}^{9}-{\left(\frac{\sigma }{r}\right)}^{6}\right]$$

Electrostatics:

$$E_{electrostatic}=\sum \frac{q_{i}q_{j}}{4\pi \epsilon_{0}r_{ij}}$$

The Universal Force Field (UFF), on the other hand, covers a broad range of elements in the periodic table, making it suitable for complex systems involving multiple element types. Studies show that UFF is highly applicable for simulating metal–CNT interfaces, particularly in capturing van der Waals interactions and charge transfer processes involving metals [39,40,41]. Thus, the UFF was applied to the remaining atoms. Its general form is as follows [39]:

$$E=E_{bonds}+E_{angles}+E_{torsions}+E_{inversions}+E_{vdW}$$

Bonds:

$$E_{bonds}=\sum \frac{1}{2}\;K_{b}(r-r_{0}{)}^{2}$$

Angles:

$$E_{angles}=\sum \frac{1}{2}\;K_{\theta }(\theta -\theta_{0}{)}^{2}$$

Torsions:

$$E_{torsions}=\sum \frac{1}{2}\;K_{\phi }(1+\cos(n\phi -\phi_{0}))$$

Inversions:

$$E_{inversions}=\sum \frac{1}{2}\;K_{\chi }(\chi -\chi_{0}{)}^{2}$$

Van der Waals:

$$E_{vdW}=\sum 4\epsilon \left[{\left(\frac{\sigma }{r}\right)}^{12}-{\left(\frac{\sigma }{r}\right)}^{6}\right]$$

Electrostatics:

$$E_{electrostatic}=\sum \frac{q_{i}q_{j}}{4\pi \epsilon_{0}r_{ij}}$$

### 2.2. Construction of Simulation System

The simulations were conducted using Materials Studio (version 8.0), a comprehensive molecular modeling software developed by Accelrys (now BIOVIA, a subsidiary of Dassault Systèmes, headquartered in San Diego, CA, USA). Materials Studio is widely used for materials science simulations, offering modules for structure building, optimization, and dynamics analysis.

The initial system was constructed using the Amorphous Cell module, which allows for the random distribution of atoms in a periodic box while mimicking molten densities. Silicon, tin, and copper atoms were randomly distributed around a (7,7) single-walled carbon nanotube using the PACKING function within Amorphous Cell. Density parameters were set to 1.8, 7.3, and 8.0 g/cm^3^ for silicon, tin, and copper, respectively, to mimic the molten state of these heteroatoms.

The (7,7) armchair SWCNT, with a diameter of ~9.5 Å and metallic properties, was chosen as a standard substrate due to its uniform lattice, moderate curvature, and widespread use in MD studies [22,24,36]. This choice isolates the effects of atomic bonding on adsorption and aggregation, avoiding chirality-induced variations. While zigzag (e.g., (10,0)) or chiral (e.g., (6,5)) CNTs may enhance localized chemisorption or asymmetric patterns, our qualitative trends are expected to persist. Future work will systematically investigate chirality effects.

The growth of heteroatoms on the CNT surface was modeled using classical MD simulations in the Forcite Dynamics module, which supports efficient integration algorithms for large-scale systems. The simulation mode employed was the NVT ensemble (constant number of particles, volume, and temperature), suitable for studying adsorption and aggregation without volume fluctuations. The CNT was kept fixed to focus on heteroatom dynamics. A time step of 0.5 fs was used, with a total simulation time of 200 ps (400,000 steps). Temperature control was achieved via the Nose–Hoover thermostat, ensuring stable thermal equilibrium and accurate representation of kinetic energy distributions during adsorption processes. During the molecular dynamics simulation, the temperatures of Si, Sn, and Cu were set to 1000, 400, and 900 K, respectively.

### 2.3. Characterization of Simulation Results

Following simulations of the different systems, orthogonal projection images at identical simulation time points were captured and exported as structure bitmaps. Additionally, perspective projection images were generated to visualize the atomic arrangement within the CNTs after simulation for each system. Finally, the two-dimensional linear density and radial distribution function of the system were computed to analyze the spatial distribution patterns of atoms and explore the intrinsic ordering characteristics of the system containing CNT inclusions.

## 3. Results

Molecular simulation results reveal that Si, Sn, and Cu atoms exhibit distinct growth behaviors on the surface of single-walled carbon nanotubes. This may be attributed to the interplay of local atomic density, thermal kinetic energy, and the constraining capacity of the substrate potential field at the statistical temperature, which arises from the “microscopic inhomogeneity” of heteroatoms.

### 3.1. Molecular Dynamics Simulation Results

#### 3.1.1. Molecular Dynamics Simulation Results of Si Atom System

**Figure 1:**
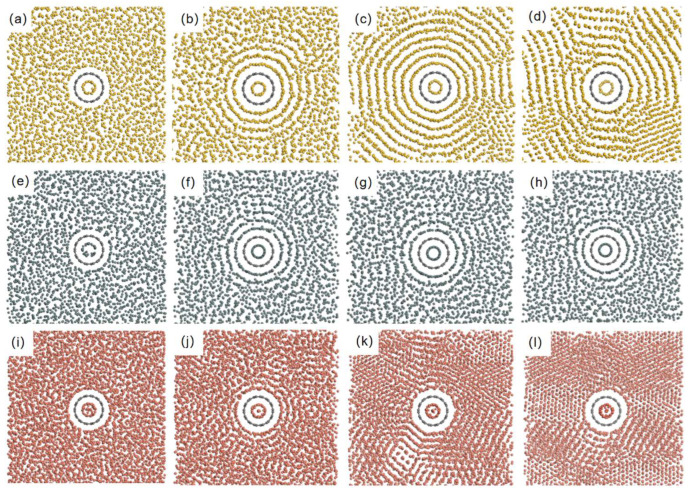
Adsorption and aggregation behaviors of (**a**–**d**) Si, (**e**–**h**) Sn, and (**i**–**l**) Cu atoms on (7,7) single-walled carbon nanotubes at simulation times of (**a**,**e**,**i**) initial state, (**b**,**f**,**j**) 12.5 ps, (**c**,**g**,**k**) 35 ps, and (**d**,**h**,**l**) 200 ps, illustrating the evolution of concentric shell structures and cluster formation.

Figure 1a–d illustrate the growth sequence of silicon atoms around the (7,7) CNT at different molecular dynamics steps. Silicon atoms grow on the CNT surface, forming characteristic concentric shells. As the simulation proceeds, three distinct annual ring-like concentric shells form outside the CNT (Figure 1b). By 70,000 steps, the Si atomic concentric shells nearly fill the lattice, with “flattening” inhomogeneity emerging within the regular layered structure. Upon simulation completion, the structure shown in Figure 1d was observed: the originally regular ring formation is significantly deformed, and only the atomic layer adjacent to the CNT retains regular concentric shells.

This phenomenon can be attributed to the inhomogeneous distribution of Si atoms in the system. At constant temperature, Si atoms in regions with relatively low local density possess higher kinetic energy. As the layered ring structure expands outward, the constraining effect of the CNT’s potential field weakens; once the atoms can overcome this potential field, these high-energy atoms break through and detach from the ring configuration, resulting in flattening and fracturing of the clusters. As the simulation continues, these discrete atoms further act as “defect templates,” disrupting the periodic arrangement of adjacent regions and causing a chain collapse of the layered structure (Figure 1c,d). Notably, the strong covalent bonding characteristic of Si promotes the formation of-ordered structures, while the rigid constraints of its bond angles and lengths amplify the destructive impact of local disturbances on the overall configuration. This behavior has been confirmed by DFT-based studies [42,43].

#### 3.1.2. Molecular Dynamics Simulation Results of Metal Atom Systems

For Sn atoms, although they also formed concentric shells (Figure 1f), the number of such structures is significantly smaller than in the Si system, with no observable damage to the concentric shells within the same simulation timeframe. Despite belonging to the same main group, the semi-metallic properties and multivalent tendency of Sn result in a weaker influence of the CNT’s potential field compared to the Si atomic system, limiting the inheritance and manifestation of the CNT’s geometric characteristics. Meanwhile, the flexibility of Sn-Sn bonds enables atoms to adapt to the local environment through valence adjustment, thereby preventing damage to the layered ring structure under conditions of “microscopic inhomogeneity.”

Cu atoms exhibited entirely distinct behaviors (Figure 1i–l). Their high diffusivity and the non-directionality of metallic bonds make it difficult to form well-defined concentric shell structures. Due to the low surface migration barrier of Cu, high-energy atoms in low-density regions readily broke through the CNT’s potential field and detach from their initial adsorption sites (Figure 1k). The cohesive energy of Cu-Cu metallic bonds (≈3.5 eV/atom) is substantially higher than the Cu-CNT interfacial energy; once atoms escaped the constraints of the potential field, they efficiently nucleated and formed clusters (Figure 1l), with these structures persisting as the simulation proceeded.

#### 3.1.3. Measurement Results of Radial Linear Density of the Systems

**Figure 2:**
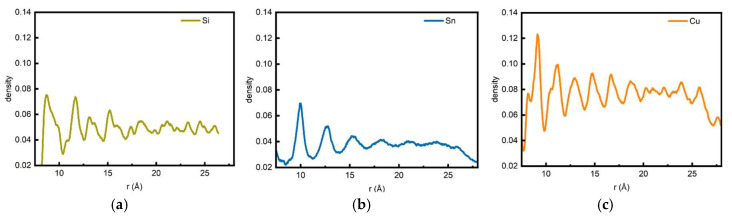
Radial linear density curves after the adsorption and aggregation of (**a**) Si, (**b**) Sn, and (**c**) Cu on (7,7) carbon nanotubes, showing the distribution of atoms relative to the carbon nanotube surface after 200 ps of simulation.

Figure 2 presents the radial linear density measurements for the three systems. The linear density curve of the Si/CNT system exhibits two prominent peaks followed by dense minor peaks. This phenomenon can be well explained as follows: in the near-stable state of the Si/CNT system, exactly two layers of the layered ring structure remain fully intact. However, due to defect accumulation induced by “microscopic inhomogeneity,” the concentric shells sustain substantial damage (Figure 1d). Although Si atoms still exhibit a concentric shell-like arrangement around the CNT, the flattened and fractured clusters significantly affect the linear density measurement results of the Si/CNT system.

In the Sn/CNT system, the linear density measurements also show good correspondence with the adsorption and aggregation behaviors observed in the simulations (Figure 1h). As seen in the simulation results (Figure 1), the Sn/CNT system forms two distinct layered structures in its near-stable state, which exactly correspond to the two main peaks in the linear density curve. Although a third layered structure formed by Sn atoms is faintly visible, Sn atoms at this distance are insufficiently influenced by the CNT’s potential field to form a clear boundary with the subsequent irregular Sn atomic arrangement. Consequently, the height of the third peak in its linear density curve decreases significantly.

In the Cu/CNT system, as typical metal atoms, Cu exhibits a linear density curve with an overall height higher than those of the Si and Sn systems. Notably, no distinct regular peaks are observed across the entire radial range, with the curve displaying a continuous, gradually decreasing trend. This characteristic can be attributed to the high diffusivity of Cu atoms and the non-directionality of their metallic bonds, which cause them to rapidly aggregate into disordered clusters on the CNT surface. These clusters break free from the constraints of the CNT’s potential field and fail to form well-defined concentric shell distributions. Expanding outward from these clusters (acting as defect templates), density fluctuations emerge in the radial direction and gradually stabilize with increasing distance from the CNT.

### 3.2. Observation of Spiral Growth Phenomenon

**Figure 3:**
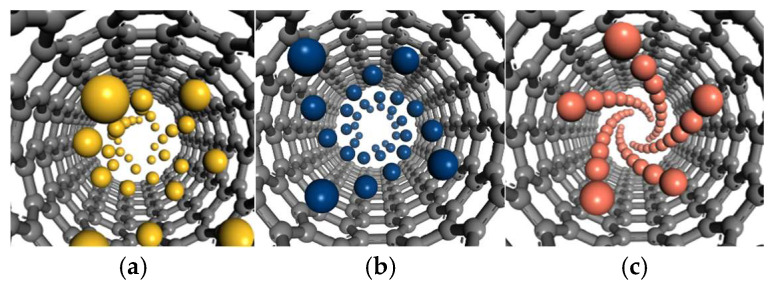
Growth of (**a**) Si, (**b**) Sn, and (**c**) Cu in a spiral pattern on the inner side of (7,7) carbon nanotubes.

Figure 3 illustrates the atomic arrangement within CNTs. It is evident that heteroatoms within the nanotubes exhibit a distinct spiral growth tendency. This phenomenon is consistent with previous findings [44], as spiral growth minimizes energy consumption and spatial occupancy—specifically, the spiral represents the energy-optimal conformation in confined spaces, facilitating a reduction in the nucleation energy barrier.

The outer layers, although heteroatoms generally tend toward spiral arrangement, increased spatial freedom and initial clusters (e.g., Cu spherical nuclei, Si segments), acting as “defect templates” disrupt spiral periodicity, significantly impairing the regularity of spiral atomic chains.

Within the CNT interior, the confinement effect of the closed cavity significantly modulates the spiral growth behaviors of Si, Sn, and Cu atoms via geometric constraints and potential field regulation (Figure 3). However, in contrast to metal atoms, while Si atoms within the nanotube exhibit a spiral tendency induced by geometric constraints, the strong directionality of Si-Si covalent bonds and the rigidity of their bond lengths and angles (≈109.5°) hindering adaptation to the CNT’s curvature stress. Consequently, under the overall spiral arrangement trend, frequent kinks and flattening phenomena occur (Figure 3a).

For Sn atoms, which belong to the same main group as Si, their semi-metallic bonds exhibit unique flexibility, which relieves curvature stress within the nanotube. Under the influence of the strong potential field in the closed space, distinct spiral growth is observed (Figure 3b). Similarly, the non-directional metallic bonds between Cu atoms enable good adaptation to the CNT’s geometric characteristics, forming a self-consistent, ordered spiral structure (Figure 3c).

Given the small electronegativity difference between Si and C, covalent compounds readily form. This leads to further reinforcement of geometric constraints by Si-C covalent interactions on the inner surface of the CNT, which also accounts for the frequent occurrence of distortions—such as kinks and flattening—under the spiral growth trend.

On the contrary, Sn and Cu, as metallic elements, rarely form compounds with C. This largely avoids additional constraints imposed by bonding between heteroatoms and the CNT, enabling them to ultimately form self-consistent, ordered, and regular spiral structures.

Additionally, differences in the number of spiral chains and spiral angles formed by Sn and Cu within CNTs are notable. The cylindrical curved inner surface of the CNT creates a strong confined geometric potential field, where its chirality and C-C bond length define the geometric framework for atomic stacking.

For Sn atoms, their valence electrons exhibit both partial locality and delocalization. While Sn-Sn bonds are more flexible than Si-Si covalent bonds, they retain weak bonding directionality. Furthermore, Sn has a relatively large atomic radius (≈141 pm), so only four stacking units can be accommodated within the confined circumference, corresponding to four spiral chains. This flexibility is partly due to the longer Sn-Sn bond lengths in β-tin, which are approximately 3.02 Å for the nearest neighbors and 3.18 Å for the next-nearest neighbors (compared to the Si-Si bond length of about 2.35 Å in diamond-structured silicon) [45,46]. These longer bonds indicate weaker interatomic interactions for Sn, allowing greater adaptability to curvature stress while maintaining some directional character. The weak directionality of semi-metallic bonds restricts the tilting freedom of Sn atoms when adapting to surface curvature—an excessively sharp spiral angle would generate stress due to directional bond constraints. Thus, the spiral angle of Sn is gentler in balancing bonding stress and spatial adaptability.

In contrast, Cu atoms rely on non-directional metallic bonds, with fully delocalized valence electrons forming an “electron sea,” rendering interatomic connections free from bond angle restrictions. Moreover, Cu has a smaller atomic radius (≈128 pm), and each stacking unit occupies a “narrower” circumferential space, allowing five stacking units to be accommodated within the same circumference (corresponding to five spiral chains). The unconstrained nature of metallic bonds enables Cu atoms to tilt freely with the nanotube’s curvature: a sharper spiral angle allows closer conformity to the curved surface, and the “lubricating” effect of delocalized electrons buffers curvature stress. Consequently, Cu exhibits a relatively larger spiral angle to achieve the energy-optimal conformation in the confined space.

### 3.3. Analysis of Heterogeneous Atom Adsorption and Aggregation Mechanism Based on Radial Distribution Function

**Figure 4:**
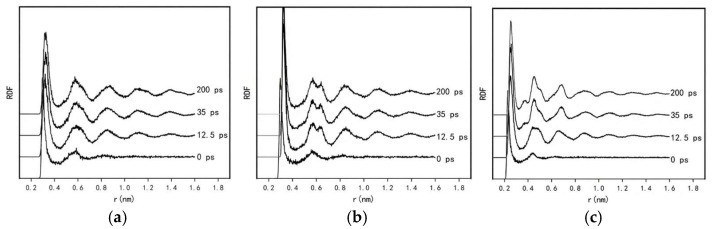
Radial distribution function (RDF) curves of the composite system of (**a**) Si, (**b**) Sn, and (**c**) Cu with (7,7) carbon nanotubes.

In the Si atomic system (Figure 4a), a clear trend is observed as the simulation progresses: the peak height of the first main peak decreases with obvious broadening, indicating local distortion of the short-range ordered structure in the Si system. This corresponds to the continuous layered ring-like aggregation behavior of Si atoms induced by CNTs. Meanwhile, the second peak increases slightly and sharpens over the simulation, signifying enhanced regularity of atomic arrangement in the medium-range order. These observations are consistent with our simulation results. The evolution of aggregation behavior in the Si system arises from the dynamic competition between Si-Si covalent bonds and the potential field constraints of CNTs. In the initial stage, covalent bonds dominate the aggregation process, driving Si atoms to form short-range ordered ring-like layered structures; the enhancement in the second peak here is attributed to the initial formation of medium-range structures. As the growth of layered ring structures approaches its limit and defect templates accumulate continuously, short-range order weakens, yet the medium-range structure remains stable through hierarchical stacking. This phenomenon highlights that the strong covalent bonding of Si atoms not only promotes the formation of initial ordered structures but also leads to structural instability in later stages due to poor disturbance resistance.

In the Sn system (Figure 4b), the height of the first peak increases. This can be attributed to the flexibility of Sn-Sn semi-metallic bonds, which balances the stress exerted by the CNT. These bonds exhibit both partial covalent bond directionality and metallic bond delocalization, allowing valence electrons to adapt to the nanotube’s potential field through flexible adjustments. Atoms form more ordered adjacent stacking via bond length fine-tuning, resulting in a significant enhancement in short-range atomic arrangement regularity. The second peak increases slightly and splits with a right shoulder, which is interpreted herein as a manifestation of phase transition competition, reflecting the coexistence of two distinct atomic coordination environments in the medium range. Under the interplay of “bonding flexibility-geometric adaptation” in the Sn system, Sn atoms near the CNT form tightly packed ring-like layered structures. In contrast, Sn atoms farther from the nanotube experience insufficient potential field stress to form layered ring structures; instead, they adopt a new coordination structure through dynamic adjustments, leading to the splitting of the second peak into a right shoulder.

In the Cu system (Figure 4c), the variation characteristics of the radial distribution function (RDF) curve are more complex, with the height of the first main peak decreasing initially and then increasing. During the early stages of the simulation, Cu atoms form a sparse adsorption layer on the CNT surface. The layered structure remains indistinct with no effective stacking, and the atomic distribution is highly random, resulting in a broadened distribution of adjacent atomic distances. This leads to a decrease in intensity and an increase in width of the first RDF peak. As the simulation proceeds, Cu atoms, via their high diffusivity, break free from the CNT’s potential field constraints, detach from their initial adsorption sites, and rapidly aggregate into disordered clusters. Within these clusters, atoms achieve close packing through the delocalization of metallic bonds, with the distribution of adjacent distances concentrating around the bond length of crystalline Cu (≈2.55 Å). This concentration causes the intensity of the first peak to recover. Additionally, in the Cu system, the second peak increases in intensity and splits with a left shoulder at approximately 3.6 Å, which also indicates the presence of two distinct coordination environments in the medium range. Specifically, as aggregation proceeds, two structural features coexist: amorphous coordination clusters that are prevalent under the CNT’s potential field, gradually forming face-centered cubic crystalline structures.

## 4. Discussion

Research on interfacial behaviors and underlying mechanisms represents a promising avenue for the continued development of nanocomposites, particularly those based on CNT/matrix systems. Due to limitations in experimental techniques and the challenges associated with direct observation at the atomic scale, molecular simulation methods have become increasingly valuable for investigating the microscopic interactions between dissimilar phases in composite materials. Although studies on interfacial systems in semiconductor and metal matrix composites—important representatives of nanocomposite applications—have been reported, these investigations are often fragmented and isolated, failing to fully capture the distinct characteristics and differences in adsorption and agglomeration behaviors induced by CNTs across various matrix materials.

To address these limitations, this study employed classical molecular dynamics simulations to investigate, at the atomic level, the adsorption and agglomeration processes of silicon, tin, and copper atoms on CNTs. The simulations revealed the formation of circular layer structures and spiral growth configurations of heterogeneous atoms under the influence of the CNT potential field. Based on these findings, we quantitatively analyzed the linear density and radial distribution function of the nanocomposite systems (Table 1).

The simulation and analysis results demonstrate that the adsorption and agglomeration behavior of matrix atoms results from the dynamic coupling between defect templates induced by “microscopic inhomogeneity” and variations in atomic bonding characteristics under the CNT potential field. Because real atomic arrangements cannot be perfectly uniform, the circular layer structures and spiral tendencies of heterogeneous atoms on CNTs are often imperfectly expressed. Specifically, the strong covalent bonds of Si (valence +4, radius ≈ 117 pm, electronegativity ≈ 1.90) facilitate the formation of initially ordered circular layers; however, their rigidity amplifies local disturbances, leading to structural instability in regions farther from the CNTs. In contrast, the semi-metallic bonds of Sn (valence +4, radius ≈ 141 pm, electronegativity ≈ 1.96) regulate curvature stress through valence flexibility, thereby maintaining the stability of circular layer structures. Meanwhile, the high cohesive energy associated with Cu’s (valence +2, radius ≈ 128 pm, electronegativity ≈ 1.90) metallic bonds promotes rapid clustering, which overcomes the constraints imposed by the CNT potential field and suppresses the formation of ordered circular layer structures.

In this work, interfacial energy is discussed qualitatively based on observed atomic configurations. Quantitative evaluation of interfacial energies would require first-principles calculations such as density functional theory (DFT), which will be considered in future studies.

Overall, our simulation results are validated by experimental studies. For instance, a layered structure of Si was observed on CNTs in the experiment [47]. Experimental HRTEM of Sn particles on CNTs via single-atom-to-cluster method shows layered particle distributions consistent with stable shells [48]. Experimental study of Cu-CNT interfaces with bond formation and clustering is consistent with metallic bond effects [49].

## 5. Conclusions

This study provides a comprehensive molecular dynamics analysis of the adsorption and aggregation dynamics of Si, Sn, and Cu atoms on (7,7) SWCNTs, revealing how atomic bonding characteristics dictate nanoscale structural evolution over a 200 ps simulation period. Unlike prior isolated investigations, our unified framework highlights distinct behaviors driven by covalent, semi-metallic, and metallic bonding, offering quantitative insights into interfacial ordering and disorder.

For Si atoms, characterized by rigid covalent bonds, initial aggregation forms up to three concentric shells within the first 12.5 ps (25,000 simulation steps), with shell radii expanding outward under the SWCNT potential field. By 35 ps (70,000 steps), these shells begin to flatten due to local density fluctuations, and, at simulation end (200 ps), only the innermost few layers remain intact, as evidenced by the linear density curves. The RDF curves analysis further quantifies this order-disorder transition, with a sharp first peak at around 2.35 Å diminishing for outer shells, reflecting how bond rigidity amplifies defects and leads to chain-like collapse.

In contrast, Sn atoms, with flexible semi-metallic bonds, form a stable configuration of two well-defined concentric shells throughout the simulation, corresponding to the two main peaks in the linear density curve, and a faint third peak fails to fully materialize due to the reduction in CNT’s potential field. RDF curves display broader peaks, underscoring valence adaptability that mitigates microscopic inhomogeneities, resulting in no observable damage as the simulation progressed. Cu atoms, governed by non-directional metallic bonds with high diffusivity and low migration barrier, rapidly form disordered clusters by 35 ps without discernible shells. The linear density profile lacks distinct peaks, instead showing diffuse clustering beyond 15 Å, while RDF curves confirm preferential Cu-Cu nucleation over CNT templating.

Due to the strong potential field of CNTs and the restricted degrees of freedom, heterogeneous atoms inside CNTs tend to adopt a spiral growth pattern. However, Si atoms exhibit kinking and flattening, which can be attributed to the strong rigidity of Si-Si covalent bonds (bond angle ~109.5°) and the tendency of Si to form covalent compounds with carbon, further enhancing structural constraints. In contrast, Sn and Cu atoms, which form metallic bonds with themselves and do not react with C, display distinct spiral growth. Moreover, Sn, with its weaker directionality of valence electrons and larger atomic radius (~141 pm), forms four gentle spiral chains within CNTs. Cu, benefiting from the non-directional nature of metallic bonds and its smaller atomic radius (~128 pm), forms five spiral chains with larger spiral angles. Both configurations represent energetically favorable structures under spatial confinement.

In summary, this study presents a comparative investigation into the interfacial adsorption and aggregation behaviors of Si, Sn, and Cu atoms on CNT surfaces. The results reveal the diversity of CNT-induced spiral growth and layered ring-structured aggregation phenomenon. These parallel simulations effectively demonstrate the significant influence of bonding interactions on composite interface systems, providing valuable insights for cross-scale composite interface design and functional applications.

## Figures and Tables

**Table 1:** This is a summary table of the key research results.

Aspect	Si	Sn	Cu
**R** **ing formation**	High initial order, but unstable	Stable limited order	Low order (clusters)
**Spiral growth**	Disrupted spirals	Ordered, 4 chains, gentler angle	Ordered, 5 chains, sharper angle
**Radial linear density**	Two main peaks	Two main peaks, while the third peak decreases	higher consecutive peaks, decreasing gradually
**RDF**	Peak broadening, medium-range stability	Peak sharpening, splitting	Peak recovery, sharpening, splitting

## Data Availability

The data that support the findings of this study are available from the corresponding author upon reasonable request.

## Abbreviations

The following abbreviations are used in this manuscript:

CNT	Carbon nanotube
SWNT	Single-walled carbon nanotube
MS	Materials Studio
RDF	Radial distribution function
